# Apoptosis-Inducing TNF Superfamily Ligands for Cancer Therapy

**DOI:** 10.3390/cancers13071543

**Published:** 2021-03-27

**Authors:** Olivia A. Diaz Arguello, Hidde J. Haisma

**Affiliations:** Department of Chemical and Pharmaceutical Biology, Groningen Research Institute of Pharmacy, University of Groningen, 9713AV Groningen, The Netherlands; O.A.D.A.Diaz@rug.nl

**Keywords:** cancer, apoptosis, TNF family, death receptors

## Abstract

**Simple Summary:**

Cancer is a complicated disease that has a significant characteristic of evading cell death (apoptosis). The induction of apoptosis in cancerous cells seems a promising procedure to use as a cancer treatment. In the family of the tumor necrosis factor (TNF) proteins, there are some ligands with the capability to induce apoptosis. Several recombinant TNF apoptosis-inducing ligands have been designed over the years, and their characteristics have been improved. This review provides an overview of the studies done in different stages of the TNF apoptosis-inducing ligands as cancer treatments and the strategies to surpass their natural limitations to improve their effectiveness.

**Abstract:**

Cancer is a complex disease with apoptosis evasion as one of its hallmarks; therefore, apoptosis induction in transformed cells seems a promising approach as a cancer treatment. TNF apoptosis-inducing ligands, which are naturally present in the body and possess tumoricidal activity, are attractive candidates. The most studied proteins are TNF-α, FasL, and TNF-related apoptosis-inducing ligand (TRAIL). Over the years, different recombinant TNF family-derived apoptosis-inducing ligands and agonists have been designed. Their stability, specificity, and half-life have been improved because most of the TNF ligands have the disadvantages of having a short half-life and affinity to more than one receptor. Here, we review the outlook on apoptosis-inducing ligands as cancer treatments in diverse preclinical and clinical stages and summarize strategies of overcoming their natural limitations to improve their effectiveness.

## 1. Introduction

Cancer is one of the leading causes of death globally; it is a complex disease that involves bypassing growth suppressors, angiogenesis, metastasis, and apoptosis evasion [1]. The most common treatment courses include chemotherapy, surgery, and radiation [2], which, in many cases, are not effective and can cause multisystemic toxicity. Therefore, the need to improve cancer treatments necessitates the development of more specific and sophisticated treatments that can target cancer cells with high selectivity without harming the healthy ones.

Apoptosis evasion is one of the main hallmarks of cancer; its dysregulation is related to overexpression of antiapoptotic genes or survival signals and downregulation/mutation of proapoptotic genes [3]. Therefore, overcoming cell death resistance by triggering the apoptotic pathways has been an area of interest in the development of cancer treatments. The study of apoptotic mechanisms has brought to the spotlight a subgroup of the apoptosis-inducing ligands. Those ligands belong to the tumor necrosis factor (TNF) family [4]; members of this family are naturally expressed by the immune system and possess tumoricidal activity [5]. These ligands can potentially be used as a cancer treatment.

This review provides an overview of the TNF family of receptors and their apoptosis-inducing ligands as cancer treatments. Additionally, we describe several engineered death receptor agonists and how gene therapy may improve the apoptosis-inducing ligands’ efficacy.

## 2. Apoptosis

**Figure 1:**
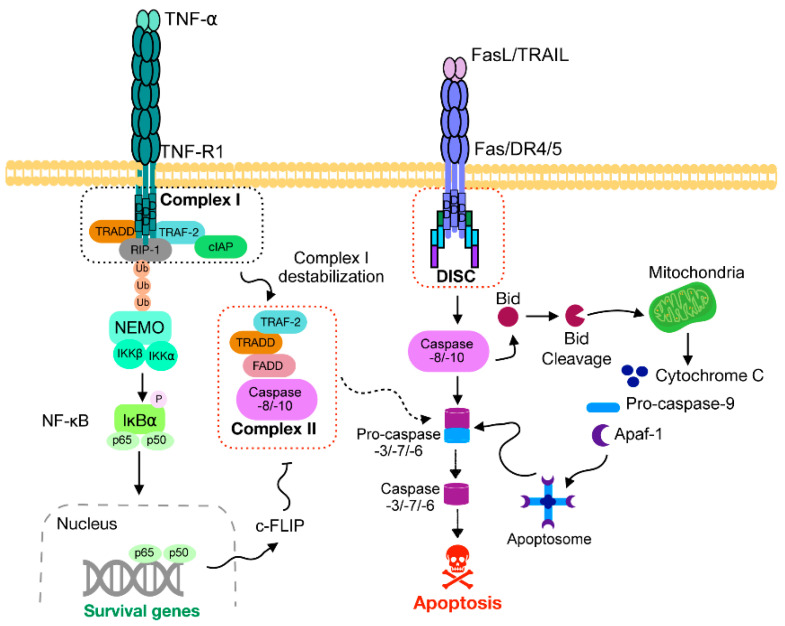
Simplified extrinsic signaling pathway from TNF-R1, Fas, DR4, and DR5.

Apoptosis is a regulated cell death process that keeps the cell population balanced in a living organism [6]. It is a mechanism that the immune system uses to prevent the accumulation of damaged or infected cells. Both the intrinsic pathway, also known as the mitochondria-dependent apoptosis pathway, and the extrinsic pathway can induce apoptosis (Figure 1) [6,7,8].

The intrinsic pathway is activated when the outer membrane of the mitochondria is permeabilized, releasing cytochrome c. Then, it binds to the apoptotic protease activating factor 1 (APAF1), which leads to the formation of the apoptosome. The apoptosome recruits caspase-9, which activates caspases-3 and -7, leading to cell death. Besides cytochrome c, the mitochondria also release the second mitochondria-derived activator of caspase (SMAC/DIABLO) and the serine protease HtrA2/Omi. These factors impede the inhibitor of apoptosis proteins (IAPs), boosting the induction of apoptosis [6,9]. The mitochondrial outer membrane permeabilization (MOMP) may occur because of different factors such as the lack of growth factors, cytokines, or hormones; DNA damage; endoplasmic reticulum (ER) stress; toxins; radiation; and viruses [10].

The extrinsic pathway’s activation requires the stimulation of death receptors (DRs); these are transmembrane receptors that belong to the TNF receptor superfamily [8]. They have a death domain (DD) that has a six-helical bundle fold as a structural characteristic [11]; this intracellular domain transfers the signal from the extracellular domain to the cytosol, starting the recruitment of death-inducing signaling complex (DISC) [12]. This process results in the autoactivation of procaspase-8 to caspase-8, leading to the activation of caspases-3 and -7 and resulting in apoptosis [6,13].

Independently of which pathway is activated, both lead to the activation of caspases-3, -6, and -7, which induce apoptosis [14]. Several members of the TNF superfamily were brought to the spotlight as cancer treatments because of their capability of specific apoptosis induction in transformed cells [15].

Even though the DRs are well known for their apoptosis induction characteristics, they may also trigger necroptosis. A regulated necrosis process mediated by the RIP kinase family members can be induced, in some cell lines, under certain circumstances, such as the use of caspase inhibitors [16], downregulation of cIAPs [17], and acidic extracellular pH [18]. In this review, we will focus on apoptosis.

### 2.1. Apoptosis-Inducing Ligands and Their Receptors

**Scheme 1:**
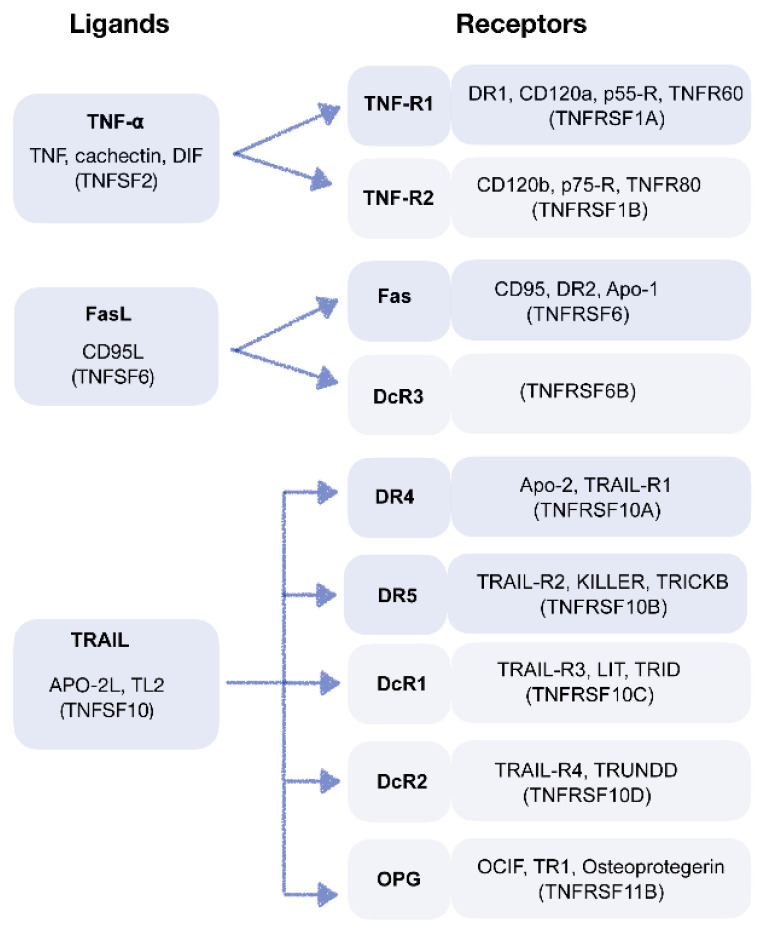
Tumor necrosis factor (TNF) apoptosis-inducing ligands and their receptors.

In recent years, members of the TNF superfamily of cytokines, TNF-α, Fas ligand (FasL), and TNF-related apoptosis-inducing ligand (TRAIL), have been studied for their apoptosis induction capability when bound to their receptors that contain a DD [19]. The most studied DRs from the TNF superfamily (TNFSFR) (Scheme 1) are TNF-R1 (DR1), CD95 (DR2, Fas), TNF-related apoptosis-inducing ligand receptor 1 (TRAIL-R1, DR4), and TRAIL-R2 (DR5) [20].

**Figure 2:**
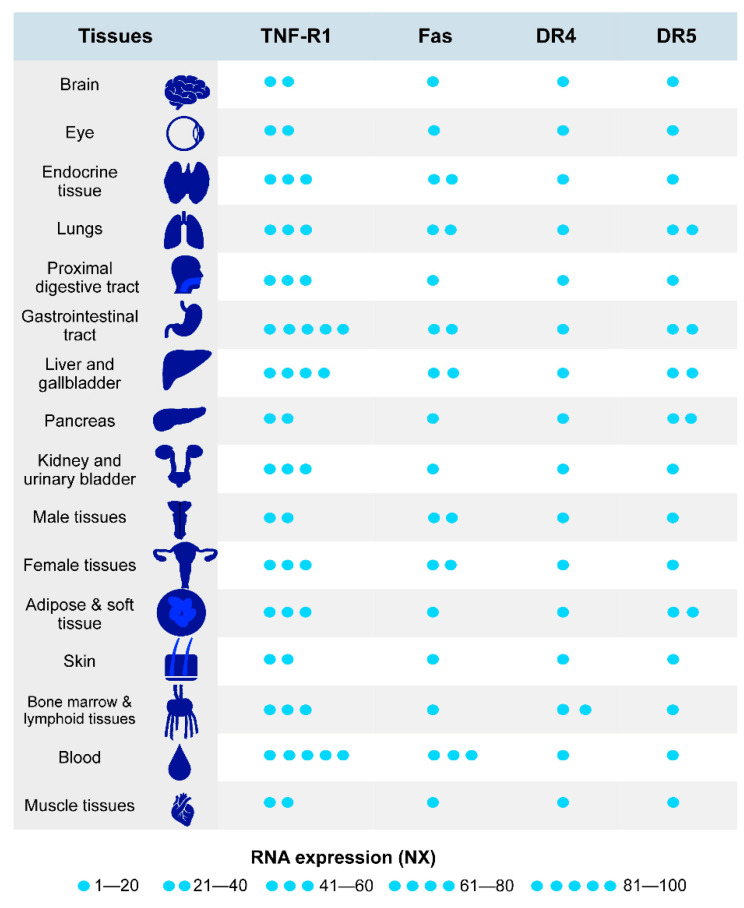
Summary of the consensus normalized expression (NX) level of each death receptor in healthy tissue. Information based on The Human Protein Atlas (https://www.proteinatlas.org, accessed on 10th March 2021), which combines the data of three transcriptomic datasheets (HPA, GTEx, and FANTOM5).

The death receptors are type I transmembrane proteins with cysteine-rich extracellular domains. The DRs have been detected in a wide variety of healthy [17,18] (Figure 2) and cancerous tissues either by assessing protein expression or RNA expression. It was found that in some cases of clear cell renal cell carcinoma (ccRCC), TNF-R1 is upregulated [19]; however, in ovarian cancer, the expression levels were similar to the ones in healthy tissues [20].

**Figure 3:**
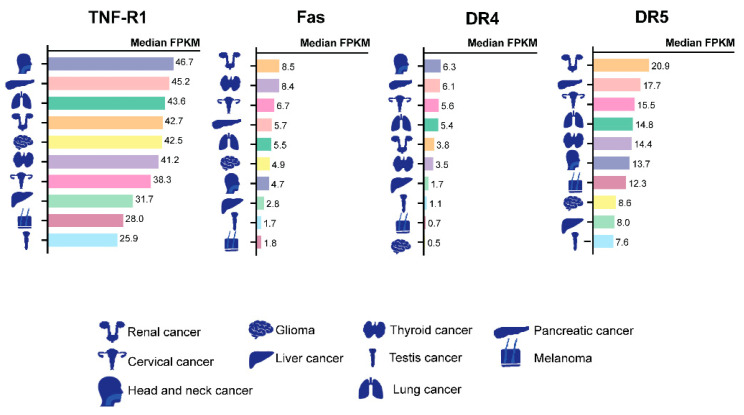
Overview of the death receptor RNA expression levels (median FPKM) in cancer patients. Information based on The Human Protein Atlas (https://www.proteinatlas.org, accessed on 13th March 2021) from the TCGA dataset.

The analysis of Fas expression revealed its downregulation in some colon carcinomas [21], lung carcinomas [22], and gynecological cancer tissues [23,24]. At the same time, DR4 and DR5 were found to be upregulated in pancreatic [25], colorectal [26], and cervical [24] cancers. It is important to remark that level of receptor expression can vary among patients with the same type of cancer (Figure 3), as Kawasaki et al. [27] and Hwang et al. [28] found; those differences can be helpful in developing biomarkers of resistance.

Interestingly, the TNF apoptosis-inducing ligands also bind to decoy receptors (DcRs). These receptors are not able to trigger the apoptosis cascade, but they can activate survival and migration signaling [29,30]; the identified DcRs are DcR1 (also known as TRAIL-R3 or TRID), DcR2 (TRAIL-R4 or TRUNDD), DcR3, and osteoprotegerin (OPG) [31]. DcR1 and DcR2 are membrane proteins, while DcR3 and OPG are soluble receptors [32].

TNF-α, FasL, and TRAIL are expressed mainly in immune cells, including granulocytes, monocytes, T cells, B cells, dendritic cells, and NK cells [33]. Most apoptosis-inducing ligands (e.g., TNF-α, FasL, and TRAIL) are transmembrane proteins that can be proteolytically cleaved and released in a soluble form. Both forms are present in noncovalent trimeric forms [34].

#### 2.1.1. Tumor Necrosis Factor-α (TNF-α)

TNF-α is a type II transmembrane protein with different biological roles: mediation of the inflammatory response, regulation of immune cells, and cytotoxicity. TNF-α binds to tumor necrosis factor receptor 1 (TNF-R1), also known as death receptor 1 (DR1), and tumor necrosis factor receptor 2 (TNF-R2); both receptors are involved in prosurvival signaling and proliferation by activating the NF-kB pathway. However, only TNF-R1 has a death domain that can trigger apoptosis through caspase cascade activation under certain conditions, such as the absence of the IAPs [35,36]. TNF-R1 can be found in basically all the cell types (Figure 3); in contrast, TNF-R2 is mainly expressed in immune cells and endothelial cells [37,38].

TNF-R1, TNF- R2, and TNF-α can be cleaved by the metalloprotease TNF-α converting enzyme (TACE or ADAM-17), resulting in the soluble forms of TNF-α (sTNF-α), sTNR-R1, and sTNR-R2 [39,40,41,42]; interestingly, sTNF-α can activate TNF-R1, but it is inferior in activating TNF-R2 [5].

Recombinant TNF-α has been studied as a cancer treatment. Unfortunately, in clinical trials, it showed systemic toxicity when administrated intravenously. Therefore, treatment directed to a specific organ or an area of the body seems to be a better option [43].

#### 2.1.2. Fas Ligand (FasL)

FasL (CD95L) is a type II transmembrane protein expressed in diverse immune cells, like B, T, and NK cells [44]. It can interact with DcR3 and Fas receptors [45]. DcR3 is a soluble secreted receptor from the TNF superfamily; when FasL binds to DcR3, it inhibits FasL/Fas apoptotic activity, acting thus as a “decoy” [46]. However, when FasL binds to Fas (also known as CD95, DR2), a type I transmembrane receptor, it starts the clustering of the receptors and recruits the Fas-associated death domain (FADD)/caspase-8 complex, leading to apoptosis [20,47]. Fas is present in a wide variety of cells (Figure 2).

Under normal circumstances, DcR3 is expressed in the brain, gastrointestinal tract, ovary, kidney, and urinary bladder, but it is difficult to detect it in serum [48,49]. However, in inflammatory diseases and cancer (e.g., breast cancer [50], renal cell carcinoma [51], and pancreatic head carcinoma [52]), overexpression of DcR3 has been identified.

A soluble form of FasL (sFasL) can be produced either by proteolytic cleavage or alternative splicing [44]. However, sFasL is not capable of activating Fas as the membrane-bound FasL does because it is not able to induce DISC formation; instead, it can activate motility signaling, and sFasL can possibly compete with FasL for receptor binding [5,44,53].

So far, recombinant FasL (rFasL) has not reached clinical trials because of the liver toxicity induced by the systemic administration of rFasL in mice [54,55].

#### 2.1.3. TNF-Related Apoptosis-Inducing Ligand (TRAIL)

TRAIL is a homotrimeric transmembrane protein type II. TRAIL is expressed on the surface of T cells, macrophages, and NK cells; its principal role is to modulate the immune response [56]. TRAIL binds to five receptors, three decoys (DcR1, DcR2, and OPG) and two death receptors (DR 4 and 5). DcR1, also known as TRAIL-R3, is a GPI-anchored protein lacking the intracellular and transmembrane domains [57], while DcR2 (TRAIL-R4) has an intracellular portion containing a truncated DD [58]; both receptors are unable to induce apoptosis after TRAIL binding. However, DcR2 activation by TRAIL triggers the NF-kB pathway [29,58]. Osteoprotegerin (OPG) is a soluble receptor that can be released by several types of tissues, including the cardiovascular system, gastrointestinal tract, lungs, kidney, bones, and immune cells [59,60]. OPG binds to TRAIL and many ligands, including another member of the TNF family, the receptor activator of nuclear factor-kB ligand (RANKL) [61]. Although DR4 and DR5 share 60% homology and both can trigger apoptosis [62], it has come to light that DR5 can trigger prosurvival, proliferative, and migration signaling in some cancer cell lines when they are treated with sTRAIL [63,64].

TRAIL is a homotrimeric protein; a zinc ion stabilizes the trimeric conformation in the Cys230 residue [65]. This conformation is essential for receptor recognition and apoptosis induction [66]. When TRAIL binds to the DRs, it induces receptor trimerization, which triggers the extrinsic apoptotic pathway [67,68]. TRAIL is known for apoptosis induction in transformed cells while sparing the nontransformed ones. This characteristic has brought TRAIL to the clinic as a cancer treatment [69].

Dulanermin is a recombinant human soluble TRAIL (sTRAIL, amino acids 114–281) protein that was tested in several clinical trials alone or in combination with chemotherapy. In phase I, it was tested as a treatment for advanced cancer [70] and metastatic colorectal cancer [71], where dulanermin was declared safe. In phase II, dulanermin was assessed as treatment of non-small-cell lung cancer (NSCLC) in combination with paclitaxel and carboplatin (PC) with or without bevacizumab (PCB), where dulanermin did not improve the efficacy of the treatment [72]. Later, in a phase III clinical trial, dulanermin was tested in combination with vinorelbine and cisplatin in the same type of cancer. There was an improvement in progression-free survival but not overall survival in the patients [73]. It is thought that the limited efficacy is related to the short half-life of dulanermin (around 1 h) [70].

A circular recombinant mutant of human TRAIL known as circularly permuted TRAIL (CPT) was developed; it consists of the N-terminal amino acids (121–135) fused with a flexible linker to the C-terminal amino acids (135–281) of TRAIL. It has a longer half-life compared to dulanermin. CPT has been tested in patients (phases Ib and II) with relapsed or refractory multiple myeloma (RRMM), where in general it was well tolerated, albeit elevation of liver enzymes was reported [74,75]. In RRMM, CPT was also tested in combination with thalidomide and dexamethasone (CPT + TD); it showed a median progression-free survival of 6.7 months in the CPT + TD group in comparison with the 3.1 months in the TD group [76].

## 3. Improving Receptor Specificity of the Apoptosis-Inducing Ligands

As mentioned before, the apoptosis-inducing ligands from the TNF superfamily interact with more than one receptor. This promiscuous interaction, in some cases, can block the activation of the apoptotic pathway [77], as in the case of TNF-α that induces apoptosis via TNF-R1 and TNF-R2 and can lead to tumor progression [40,78], and TRAIL also binds to decoy receptors (DcR1 and DcR2) that do not induce apoptosis [79]. Therefore, many TNF-α and TRAIL mutants have been engineered (Table 1) by mutating a few amino acids, thus improving the affinity towards one of their death receptors [80]. These proteins have been investigated in preclinical studies showing promising results. However, their efficacy in clinical trials remains to be demonstrated.

### 3.1. DR-Targeting Antibodies

The study of antibody-mediated therapies to treat cancer, where monoclonal antibodies (mAbs) are designed to target a specific antigen [93], such as the death receptors [94,95], has been going on for years. A well-designed mAbs can have high specificity, fewer adverse effects than apoptosis-inducing ligands, and a half-life that can last weeks [95].

Although antibody targeting of TNF-R1 as cancer therapy seems promising due to its proinflammatory signaling [96], diverse TNF-R1 antagonist antibodies (e.g., Atrosab) have been designed as treatment of rheumatoid arthritis (RA) and multiple sclerosis, among other autoimmune diseases [97,98]. Therefore, TNF blockers have been used to ameliorate the immune-related adverse events in patients undergoing cancer treatment with immune checkpoint inhibitors (ICIs) [99]. In patients with melanoma (phase Ib), it was found that the TNF inhibitors (infliximab or certolizumab) can boost the antitumor effect of ICIs (ipilimumab and nivolumab) [100].

A monoclonal antibody against murine Fas developed in the early 1990s showed liver toxicity in mice after intraperitoneal administration [101]. Later, HFE7A, a mouse anti-human Fas mAb, was designed to induce apoptosis in lymphocytes without showing liver toxicity [102]. HFE7A was able to ameliorate the symptoms of lymphadenopathy in mice by inducing apoptosis in T cells. Moreover, it induced apoptosis in synovial cells from patients with rheumatoid arthritis [103]. However, the worrisome immunogenicity of HFE7A led to the development of humanized antibody designs with improvements in pharmacokinetics and lower immunogenicity [93].

Many DR4- and DR5-targeting antibodies have been designed for cancer treatment. Mapatumumab (HGS-ETR1) is a fully human agonist monoclonal antibody (mAb) that binds with high affinity to DR4 [104]. This monoclonal antibody was tested in several clinical trials alone or with chemotherapy, showing that it was well tolerated [105]. However, there was no response in advanced solid tumors [106], non-small-cell lung carcinoma [107], and hepatocellular carcinoma [108].

Of several monoclonal antibodies designed for targeting DR5, only five reached clinical trials: conatumumab [109,110,111,112,113], lexatumumab [114,115], tigatuzumab [116,117,118], drozitumab [119,120], and LBY135 [121]; however, none of them reached phase III clinical trials. These antibodies showed a prolonged half-life in serum. Still, they failed to show an antitumor effect, which may be related to their inability to induce the oligomerization of death receptors [45]. A new generation of DR-targeting antibodies has been designed to improve bioactivity, e.g., the bispecific antibody RG7386/RO6874813 that targets the fibroblast-activation protein (FAP) and DR5 [122]. This FAP-DR5 antibody was recently tested in a phase I study in patients with advanced or metastatic tumors (NCT02558140); the results have not been published yet. Another example is the hexabodies, engineered Fc fragments that boost IgG hexamers’ formation upon binding to the membrane-bound antigen [123]. HexaBody-DR5/DR5 (GEN1029) is a mixture of two noncompeting DR5 antibodies with a hexamerization-enhancing mutation [124]; this antibody is being tested in a phase I/II trial in patients with solid tumors (NTC03576131).

### 3.2. Trimer Conformation Plays a Crucial Role in Receptor Activation

As mentioned previously, the TNF apoptosis-inducing ligands are present as trimeric transmembrane proteins that can be cleaved, resulting in soluble proteins [34]. These proteins can interact with their respective receptors but sometimes cannot activate them [125], as in the case of sFasL. Studies have shown that the re-enforcement or improvement of the trimeric conformation improves the soluble protein’s activity [125]; the most common approaches use leucine zippers, His-tagged proteins, and covalent trimerization domains [125].

The activation of Fas by sFasL requires the oligomerization of sFasL [126]; with this in mind, APO010 (MegaFasL) was designed. APO010 is a hexameric fusion protein created by the fusion of the collagen domain adiponectin to two FasL extracellular domain trimers [127]. This fusion protein has reached clinical trials (NCT00437736); the results have not been published [128].

In the case of TRAIL, different approaches have been followed to help stabilize the trimer conformation, from using a fused leucine zipper motif, the N-terminal of sTRAIL (LZ-TRAIL) [129], to using a FLAG-tag that also helps in TRAIL purification [130]. Although these TRAIL-tagged forms have stable trimeric conformations, they can form aggregates that can induce toxicity to healthy human cells [131,132]. Another option is to stabilize the trimeric conformation by covalent linkage of the monomers, creating a single-chain TRAIL (scTRAIL) protein conferring an increase in activity and decrease in aggregates [133].

### 3.3. Fusion Proteins Improve Receptor Activation and Half-Life

Fusion proteins have been used to improve the activity of the soluble form of the apoptosis-inducing ligands. For years, it has been known that soluble ligands have a binding activity differing from that of membrane-bound ligands [134]. As described previously, sFasL barely activates Fas [126], and sTRAIL can efficiently activate DR4 but is not very efficient in DR5 activation [135]. Research has shown that genetically fusing the soluble form of apoptosis-inducing ligands to Fc-domains of antibodies or single-chain variable fragments (scFv) in a fusion protein overcomes the lack of activity [133,134].

Fusion proteins are molecules with multifunctional properties depending on the moieties that conform to them [136]. A common approach is to fuse the apoptosis-inducing ligands to an scFv. The scFv is a small fusion protein that contains the variable region of heavy (V_H_) and light (V_L_) of the immunoglobulin; these two regions are bonded together by a small and flexible linker [137]. This engineered antibody keeps the full antigen-binding capacity, making it useful for cancer therapy because it can target specific antigens expressed in transformed cells [138].

#### 3.3.1. TNF-α

Several fusion proteins containing TNF-α have been designed as a cancer treatment (Table 2). A fusion protein composed of an scFv antibody from the high-molecular-weight melanoma-associated glycoprotein gp240 (ScFvMEL) and TNF-α known as scFvMEL/TNF was tested in murine models to assess the therapeutic effect, toxicity, and pharmacokinetics [139]. The results showed a therapeutic effect at a dose of 2.5 mg/kg in athymic mice with melanoma xenograft tumors; the maximum tolerated dose (MTD) was 4 mg/kg, and the terminal-phase half-life was 17.6 h after IV administration [139].

Another TNF-α fusion protein is the IL-12-L19-TNFα a triple fusion protein containing interleukin-12 (IL-12), an antibody fragment specific to fibronectin (L19) extra-domain B, and TNF-α. It was tested in vitro, where it showed cytotoxic activity in murine L-M fibroblast [143]. However, when tested in mice for biodistribution assessment, it was found that the triple fusion protein was rapidly cleared from kidneys and liver, thus failing to localize the tumor and showing no activity whatsoever. Its lack of effectiveness is related to the large size of the protein (340 kDa), which affects its biodistribution [143]. In the same study, the fusion protein L19-TNFα was tested, showing a better tumor uptake than the triple fusion protein.

The fusion protein L19-TNFα was designed for localized administration and reached clinical trials. L19 has an affinity for the EB-D domain of fibronectin, which is considered a marker of angiogenesis in cancer patients [147]. It was tested in phase I/II clinical trials as monotherapy for advanced solid cancer patients, where it showed its safety; however, it did not show an objective tumor response [145]. Later, the fusion protein safety was tested in combination with melphalan in isolated limb perfusions (ILP) in extremity melanoma patients, showing promising results [146].

#### 3.3.2. FasL

Although membrane-bound FasL can induce apoptosis, sFasL lacks the apoptotic activity, which can be regained by fusing sFasL with an scFv. Different FasL fusion proteins have been designed to improve FasL apoptotic activity. Those proteins (Table 3) have been tested in preclinical studies showing promising results; for instance, the fusion protein sc40-FasL was tested against tumor stroma in mice by intravenous administration, showing no signs of systemic toxicity and preventing the growth of FAP-positive cells [148]. Moreover, scFvRit:sFasL was studied as a treatment for B-cell leukemia. It triggered CD20 and Fas apoptotic signaling in malignant B cells in samples from patients without showing systemic toxicity in mice [149].

#### 3.3.3. TRAIL

The use of engineered antibodies in the format of an scFv is an explored approach to create TRAIL fusion proteins with high stability and the capacity to activate the apoptotic pathway [45]. The fusion of sTRAIL (20 kDa) with an scFv (30 kDa) helps to overcome the short half-life of sTRAIL because the molecular weight of the homotrimeric scFv:sTRAIL fusion protein, around 150 kDa [134,153], decreases the renal clearance and increases the time in the circulation. TRAIL has been linked to different scFvs and antibodies (Table 4) over the years. Most of the fusion proteins are in preclinical studies, like scFv-scTRAIL and CD19-sTRAIL. scFv-scTRAIL is a fusion protein composed of an scFv against the extracellular domain of ErbB2 genetically fused to three TRAIL protomers expressed as a single polypeptide chain (scTRAIL). This fusion protein showed, in vivo, an increase in the half-life and a higher apoptotic activity when compared with scTRAIL [154]. CD19L-sTRAIL is a fusion protein that contains the ligand of the human CD19 receptor (CD19L) genetically fused to sTRAIL. CD19 is a receptor expressed in B-cell precursor acute lymphoblastic leukemia. This protein was well tolerated in mice at doses between 32 fmol/kg and 3.2 pmol/kg; it also showed apoptotic activity in C19^+^ xenograft mouse models at doses in the fmol/kg range [155]. The TRAIL fusion proteins mentioned showed potential as cancer treatments.

Another TRAIL fusion protein in clinical trials is ABBV-621. It is now in phase I study as a therapy for patients with previously treated solid tumors and hematologic malignancies (NCT03082209). ABBV-621 is a fusion protein that contains an immunoglobulin G1 (IgG1)-fragment crystallizable region (Fc) portion fused to a single chain trimer of TRAIL subunits. This fusion protein was designed to maximize the clustering of TRAIL receptors [94,166].

## 4. Gene Therapy

Gene therapy may deliver proapoptotic genes to express these proteins to induce cell apoptosis [167,168]. The gene delivery can be through viral and nonviral vectors. The viral vectors possess the natural advantage of gene delivery into a wide range of host cells, promoting high transgene expression levels. The vectors can be genetically modified to alter their cell tropism, modify or remove the ability to replicate, and deliver a transgene with therapeutic properties [80,169,170,171]. Moreover, the transgene local effect can be achieved with tissue-specific or tumor-specific and inducible promoters [172]. Hence, gene therapy can be used for a local production of the apoptosis-inducing ligands, thus avoiding systemic toxicity; moreover, it can improve the ligands’ pharmacokinetics by the continuous production of the transgene [40].

Many viral vectors have been designed to deliver apoptosis-inducing ligands throughout the years, but only a few reached clinical trials, such as the adenoviral vectors TNFerade [173,174] and VB-111 [175,176].

TNFerade is a second-generation replication-defective adenovirus armed with human TNF-α cDNA. The vector includes the radiation-inducible promoter early growth response (Egf-1) upstream of the transcriptional start site of human TNF-α [177]. The Egf-1 promoter is activated by radiotherapy, which is usually applied in the tumor’s localized area; this helps regulate TNF-α’s transcription, keeping its activity restricted to a location and avoiding systemic toxicity [178].

TNFerade was used in clinical trial phases I and II to treat different types of cancers, where the patients tolerated the treatment well. However, in phase III, TNFerade was used to treat advanced pancreatic cancer but was not effective [174,177,179].

VB-111 (ofranergene obadenovec) is a replication-defective adenovirus serotype 5 vector armed with a modified murine pre-proendothelin promoter (PPE-1) and human Fas-chimera transgene. The modified PPE-1 promoter has a hypoxia-responsive factor and three copies of the endothelium-specific element that increase the specificity for angiogenic vessels—the vector was designed to target Fas-chimera transgene expression in angiogenic blood vessels to induce apoptosis [180]. In a phase I clinical trial, VB-111 was tested in patients with solid tumors, and safety and tolerability were established [175,180,181]. Then, VB-111 was then tested in patients with recurrent glioblastoma (rGBM) in a phase I/II (NCT01260506) study. The results showed that the patients primed with VB-111 alone and then treated with bevacizumab (a monoclonal antibody against the vascular endothelial growth factor (VEGF)) and VB-111 had significantly better survival and progression-free survival [182].

Later, VB-111 was tested in combination with bevacizumab in a phase III study (NTC02511405), where it did not show efficacy. It is essential to consider that the treatment setting was different from the previous phase I/II study. In this trial, the patients had a combinational treatment of VB-111 and bevacizumab. Coughesy et al. thought that the lack of effectiveness in this trial was because bevacizumab antagonized VB-111 [176]. VB-111 is still a matter of study in combination with different drugs as treatment for colorectal cancer (NCT04166383) and ovarian cancer (NCT03398655).

The adenovirus vectors can also be engineered as oncolytic viruses; they can replicate selectively in cancer cells and kill them, releasing new viral particles that can infect neighboring and distant cancer cells [172]. An example of an oncolytic adenoviral vector is the H5CmTERT-Ad/TRAIL. This vector has six copies of hypoxia-responsive elements (HER) upstream of a cancer-specific modified human telomerase reverse transcriptase (5CmTERT) promoter, and it is armed with sTRAIL (114–281 aa). H5CmTERT-Ad/TRAIL was tested in subcutaneous and orthotopic xenograft models of glioblastoma. It showed the capability to replicate efficiently in normoxic and hypoxic conditions and a strong antitumor effect potentiated by sTRAIL, which helps with the viral distribution and apoptotic induction in TRAIL-resistant glioblastomas [183]. H5CmTERT-Ad/TRAIL is a good candidate for further investigation as glioblastoma treatment.

Another example of an oncolytic vector is the NDV/Anh-TRAIL [184]. This vector was designed using the Newcastle disease virus (NDV) from the Anhinga strain, a single-stranded nonsegmented negative-sense RNA virus with natural oncolytic activity. This characteristic gives the NDV the capability to replicate in interferon (IFN)-deficient cells, i.e., cancer cells [185]. NDV/Anh-TRAIL contains sTRAIL, which improves the apoptotic capacity of the vector. The vector was tested as a treatment for hepatocellular carcinoma in vivo and in vitro. It affected the cell viability of HepG2 cells, and it was well tolerated without significant toxicity in the hepatocellular carcinoma mouse model leading to tumor regression [184].

In the case of TRAIL, no gene therapy has reached clinical trials yet. However, several viral vectors have been designed to increase TRAIL effectiveness and avoid repeated administration [69]; those vectors are being studied in preclinical models (Table 5). The most common viral vector used is the adenovirus (Ad). It can be engineered as a replication-defective vector like the Ad-scFv425:sTRAIL; this vector contains the anti-EGFR single antibody chain fragment (scFv425) fused to sTRAIL. In an in vitro test, the vector showed potent apoptotic activity in transformed infected cells and noninfected EGFR-positive cancer cells.

Additionally, Ad-scFv425:sTRAIL was tested in an established renal carcinoma xenograft in nude mice. After an intraocular administration of 10^10^ viral particles (vp), the tumor size decreased by around 90%. The fusion protein was detected in plasma 60 days after infection with a concentration of around 200 μg/mL. It did not show liver toxicity, thus proving to be safe for systemic administration [196].

## 5. Conclusions

As a cancer treatment, induction of cell death by apoptosis appears an interesting pathway to follow. In search of apoptosis-inducing ligands, targeting the death receptors (DRs) from the TNF superfamily seems the right approach. The cytokines from the TNF superfamily, TNF-α, FasL, and TRAIL, can induce apoptosis by activating the extrinsic pathway when they bind to DR1, DR2, DR4, or DR5 [216].

Although TNF-α was identified as promising cancer therapeutic and several agonists were developed for that purpose, TNF-α antagonists have a greater value in treating inflammatory and autoimmune diseases (rheumatoid arthritis) [96,217]. Recently, in a phase Ib study, it was found that the TNF blockers (infliximab or certolizumab) helped to mitigate the immune-related adverse events in melanoma patients undergoing treatment with immune checkpoint inhibitors (ICIs) while enhancing their antitumor effect [100]. Out of the TNF apoptosis-inducing ligands, TRAIL is a promising anticancer agent, mainly because of its characteristic of selectively inducing apoptosis in cancer cells while sparing the nontransformed cells. However, TRAIL’s short half-life has been a limitation for its use in the clinic.

In clinical trials, some of the recombinant death ligands and agonists developed as cancer treatments showed limited activity and, in some cases, systemic toxicity [43,70]. Therefore, gene therapy may be a useful tool to improve the activity of the apoptosis-inducing ligands and eliminate systemic toxicity.

The use of viral vectors can improve the apoptosis-inducing ligands’ effectiveness; using tumor-specific promoters can give a localized effect, thus averting systemic toxicity. The transgene’s continuous production can help overcome the short half-life of the ligands and avoid the need for multiple administrations. For instance, the use of Fas agonists in a systemic administration can lead to liver toxicity, which may be prevented by localized administration with adenoviral vectors and tumor-specific promoters. The drawback of TRAIL’s short half-life can be overcome using an adenoviral vector, as Bremer et al. demonstrated [196].

Although TNF apoptosis-inducing ligands have been designed as cancer therapies and analyzed in preclinical studies showing promising results, only a few have reached clinical trials. The majority of them faltered in the first stages of the studies, showing a lack of effectiveness. Therefore, the use of gene therapy as a tool to improve the pharmacokinetics and meet the need for better biomarkers of resistance, which help screen and select the target population to design tailored anticancer approaches from single to combination agents, may help overcome the lack of effectiveness during the clinical trials. Additionally, the TNF apoptosis-inducing ligands’ effectiveness may also be boosted by using them in combination with other anticancer therapeutics.

Considering that the gene therapy techniques are being developed and improved to such an extent that vaccines against SARS-CoV-2 were developed in record time using Ad5 [218] and lipid nanoparticles [219,220] as vectors, we expect a major step forward in the field of cancer gene therapy based on all the developments over the last few decades.

## Figures and Tables

**Table 1:** TNF-α and TRAIL mutants.

Based on	Protein	Format	Modification	Affinity	Ref.
TNF-α	Mutant 471		1-7del + P8R/S9K/D10R		[81]
	mutant R32w		R32w	TNF-R1	[82]
	Mutant M3		1-7del + S52I, Y56F	TNF-R1	[83]
	RGD-V29		includes cell adhesive sequence (R4, G5, D6) + R29V	TNF-R1	[84]
	rmhTNF		1-7del + P8R/S9K/D10R/L157F		[85]
TRAIL	TRAIL.R1-5	TRAIL (aa 95–281)	Q193S/N199 V/K201R/Y213 W/S215N	DR4	[86]
	4c7	TRAIL (aa 114–281)	G131R/R149I/S159R/ N199R/K201H/S215D	DR4	[87]
	rTRAIL ^DR4^		S159R	DR4	[88]
	FLAG-Apo2L.DR5–8 Trp-213;	TRAIL (aa 96–281)	Y189 N/R191 K/Q193R/H264R/ I266L/D267Q	DR5	[89]
	DHER	TRAIL (aa 114–281)	D269H and E195R	DR5	[90]
	DR5-A	TRAIL (aa 114–281)	Y189 N/R191 K/Q193R/H264R/ I266L/D267Q/D269H	DR5	[91]
	DR5-B	TRAIL (aa 114–281)	Y189 N/R191 K/Q193R/H264R/ I266L/D269H	DR5	[91]
	TRAIL-Mu3	TRAIL (aa 114–281)	aa 114–121 (VRERGPQR) were replaced by RRRRRRRR	DR4 and DR5	[92]

**Table 2:** TNF-α fusion proteins.

Name	Fusion Domain	Format	Ref.
anti-FAP-TNF	FAP-positive tumor stroma	humanized anti-FAP Fab + TNF	[140]
sFv23/TNF	HER2/neu	scFv23 + TNF	[141]
MFE-23:TNF-α	carcinoembryonic antigen (CEA)	scFvMFe-23 + TNF	[142]
IL-12-L19-TNF-α	T-cell-stimulating factor and scFv (L19) against the EDB domain of fibronectin	Triple fusion protein: IL-12 + scFvL19 + TNF-α	[143]
scFvMEL/TNF	gp240 antigen on human melanoma cells	scFvMEL + TNF-α	[139,144]
L19-TNF *	EDB domain of fibronectin	hmAb L19 + TNF-α	[145,146]

* L19-TNF is in clinical trials.

**Table 3:** FasL fusion proteins.

Name	Fusion Domain	Format	Ref.
sc40-FasL	scFv against fibroblast activation protein (FAP)	CD152 + FasL	[148]
scFvCD7:sFasL	scFv against CD7 (T-cell leukemia-associated antigen)	scFv40 + FasL	[150]
scFvRit:sFasL	scFv against CD20 (Rituximab)	scFvCD7 + sFasL	[149]
CTLA-4-FasL	Extracellular domain of receptor CTLA4 (B/)		[151]
cc49scFv-FasLext	scFv against human tumor-associated glycoprotein (TAG-72)	scFvRituximab + sFasL	[152]

**Table 4:** TRAIL fusion proteins.

Name	Target Antigen	Combination	TRAIL Format	Ref.
scFvC54:sTRAIL	EGP2	-	sTRAIL	[156]
scFv425:sTRAIL	EGFR	Iressa	sTRAIL	[157]
scFv425:sTRAILmR1-5	EGFR	Cisplatin, valproic acid	DR4-specific sTRAIL mutant	[158]
scFv-scTRAIL	ErbB2	-	three sTRAIL monomers (aa 95–281)	[154]
Anti-MCSP:TRAIL	MCSP	Rimcazole	sTRAIL	[159]
scFv:G28-TRAIL	CD40	-	TNC-TRAIL (95–281);	[160]
scFv:CD70-TRAIL variants	CD27	-	TNC-sTRAIL monomer (aa 99–281). wt, DR4, and DR5-specific	[161]
scFvM58-sTRAIL	MRP3	-	sTRAIL	[162]
CD19L-sTRAIL	CD19	Radiation	sTRAIL (aa 114–281)	[163]
scFv62-TRAIL	Kv10.1	Doxorubicin	Full-length TRAIL	[164]
ss-TR3	Mesothelin	-	Covalent linked-TRAIL trimer (Monomer aa 91–281)	[165]
ABBV-621	Human IgG1-Fc	Venetoclax (DLBCL, AML only), FOLFIRI + bevacizumab (KRAS-mutant CRC)	scTRAIL-RBD	NCT03082209

TCN, trimerization domain; DLBCL, diffuse large B-cell lymphoma; AML, acute myeloid leukemia; CRC, colorectal cancer; RBD, receptor-binding domain.

**Table 5:** Viral vectors armed with TRAIL protein for cancer treatment.

**Vector**	**Type**	**TRAIL format**	**Target/Aim**	**Ref.**
Ad/TRAIL-F/RGD	Replication-defective adenovirus		Pancreatic cancer NSCLC	[186] [187]
Ad/TRAIL-E1	Oncolytic adenovirus		NSCLC Colon cancer Gastric cancer	[188,189,190]
Ad-TRAIL	Replication-defective adenovirus		NSCLC Glioblastoma Lung cancer cells AML	[191,192] [193] [194]
Ad-stTRAIL	Replication-defective adenovirus	SS-ILZ-TRAIL (114–281 aa)	Solid tumors	[195]
Ad-scFv425:sTRAIL	Replication-defective adenovirus	scFv against EGFR + sTRAIL	Renal carcinoma	[196]
Ad-IU2	Oncolytic adenovirus	Full-length TRAIL	Prostate cancer	[197]
AAV-hTERT-TRAIL	Adeno-associated virus		HCC	[198]
AAV/TRAIL	Adeno-associated virus		HNSCC	[199]
Ad/gTRAIL	Replication-defective adenovirus	GFP-TRAIL	Glioma	[200]
AAV-TRE-TRAIL and AAV-Tet-On	Adeno-associated virus	soluble TRAIL	Breast cancer	[201]
ZD55-TRAIL-(IETD)-Smac	Oncolytic adenovirus	TRAIL-(IETD)-Smac	Hepatoma	[202]
Ad-KDRscFv:sTRAIL	Replication-defective adenovirus	scFv against VEGF + sTRAIL (114–281 aa)	Solid tumors	[203]
Ad-TRAIL-MRE	Replication-defective adenovirus		Bladder cancer	[204]
			Uveal melanoma	[205]
			Breast cancer	[206]
			Esophageal cancer	[207]
			Prostate cancer	[208]
			Lung cancer	[209]
Ad-ΔB/TRAIL plus	Oncolytic adenovirus		HCC	[210,211]
AAV9-NSE-sTRAIL	Adeno-associated virus		Glioblastoma	[212]
CD55-TRAIL-(IETD)-MnSOD	Oncolytic adenovirus	TRAIL-(IETD)-MnSOD	Lung cancer	[213]
NDV/Anh-TRAIL	Newcastle disease virus/oncolytic virus	Soluble TRAIL	HCC	[184]
rAAV2-sTRAIL 95-281	Adeno-associated virus		Solid tumors	[214]
H5CmTERT-Ad/TRAIL	Oncolytic adenovirus	sTRAIL (114–281 aa)	Glioblastoma	[183]
Oncopox-trail	Oncolytic poxvirus		Lung cancer	[215]
